# The South Georgia Medical Association

**Published:** 1894-02

**Authors:** 


### THE SOUTH GEORGIA MEDICAL ASSOCIATION.

We have just been advised of the organization of the South Georgia Medical Association.

The formation of such a society in South Georgia has been the subject of discussion for quite a long time, but nothing toward effecting a permanent organization was done until a meeting held in Helena, Ga., a few days ago, resulted in the launching of the Association with the above name.

The meetings are to be held in Helena on the first Tuesdays of March, June, September and December. The following officers were elected for the ensuing year:

President, Dr. G. W. Blanton, Chauncey, Ga.

First Vice-President, Dr. H. J. Smith, McRae, Ga.

Second Vice-President, Dr. J. D. Herman, Eastman, Ga.

Secretary and Treasurer, Dr. Joseph A. Ester, Eastman, Ga.

Graduates of the regular school of medicine are eligible to membership, and the doctors of the southern section of the State are cordially invited to affiliate with this Association. The aims of this new organization are not in any way antagonistic to that great and influential body, the Medical Association of Georgia, but it is a helper, an offshoot, as it were, which will prove of material interest to each association.

We are heartily in favor of the formation of such associations in various sections of the State, for the great good which is accomplished by the intermingling of professional brethren, the exchange of ideas, the report of interesting cases, and last, but not least, the good fellowship which always prevails and the social intercourse always attendant upon such meetings will prove of inestimable benefit to any man becoming a member.

We hope to have the pleasure of publishing the transactions of this promising body after each meeting. D. H. H.

